# Expression of dedifferentiation markers and multilineage markers in U251 glioblastoma cells with silenced EGFR and FGFR genes

**DOI:** 10.3892/ol.2013.1685

**Published:** 2013-11-18

**Authors:** JUE XIE, YUE-HUI MA, MING WAN, REN-YA ZHAN, YONG-QING ZHOU

**Affiliations:** 1 Department of Blood Transfusion, The First Affiliated Hospital, College of Medicine, Zhejiang University, Hangzhou, Zhejiang 310003, P.R. China; 2 Department of Neurosurgery, The First Affiliated Hospital, College of Medicine, Zhejiang University, Hangzhou, Zhejiang 310003, P.R. China

**Keywords:** small interfering RNA, epidermal growth factor, bFGF, U251 glioblastoma cell, CD133, GFAP, TUBB4, MBP

## Abstract

Epithelial growth factor (EGF) and basic fibroblast growth factor (bFGF), and their receptors, epithelial growth factor receptor (EGFR) and bFGF receptor (bFGFR), are frequently overexpressed in high-grade gliomas. In the present study, the EGF and bFGF levels in U251 glioblastoma cell culture supernatants were determined by ELISA, and enhanced green fluorescent protein (EGFP)-labeled recombinant lentiviral expression vectors with small interfering RNA targeting the EGFR and bFGFR genes were constructed. The mRNA expression levels of EGFR, bFGFR, cluster of differentiation (CD)133, glial fibrillary acidic protein (GFAP), tubulin-β3 (TUBB3) and myelin basic protein (MBP) were determined using quantitative polymerase chain reactions in U251 cells prior to and following silencing of the EGFR and/or bFGFR genes. Prior to silencing, the U251 cells secreted EGF and bFGF, and expressed EGFR, bFGFR, CD133, GFAP, TUBB3 and MBP mRNA. Subsequent to silencing the EGFR and/or bFGFR gene, CD133 mRNA expression decreased and GFAP and TUBB3 mRNA expression increased. Silencing the EGFR and FGFR genes acted synergistically to downregulate CD133 expression. The downregulation of CD133 mRNA expression and the upregulation of GFAP and TUBB3 mRNA expression were not significantly different when blocking the EGFR and FGFR pathways. These results indicate that autocrine or paracrine EGF and/or FGF mechanisms exist in U251 cells. Knocking down the EGFR and/or FGFR genes downregulates CD133 mRNA expression and facilitates glial and neuronal differentiation in U251 cells.

## Introduction

Previous studies have demonstrated that the overexpression of basic fibroblast growth factor (bFGF) and epithelial growth factor (EGF), and their receptors, epithelial growth factor receptor (EGFR) and bFGF receptor (bFGFR), occurs frequently in high-grade gliomas and glioblastomas (1–3). These studies indicated the existence of autocrine and/or paracrine loops of EGF-EGFR and FGF-FGFR in the glioblastoma microenvironment. Glioblastomas are the most malignant neuroepithelial tumors of the brain, and typically express glial fibrillary acidic protein (GFAP).

An increasing number of studies have indicated that glioblastomas develop from multipotent cancer stem cells, which are a small subset of tumor cells possessing the capacity of self-renewal and the ability to proliferate into heterogeneous lineages of cancer cells under specific environmental stimuli (4–6). It has been observed that EGF and bFGF are critical components of the culture medium for cancer stem cells and human embryonic stem cells, and are necessary for maintaining these stem cells in an undifferentiated state (7–9). These findings indicate that EGF/EGFR and FGF/FGFR may be responsible for a microenvironment that maintains glioblastomas and glioblastoma-derived stem cells in an undifferentiated state.

In the present study, the functions of EGF and bFGF were inhibited using small interfering RNA (siRNA) to interfere with their receptors, and the expression of the markers for stem cells [cluster of differentiation (CD)133], astrocytes (GFAP), neurons [tubulin-β3 (TUBB4)] and oligodendrocytes [myelin basic protein (MBP)] were analyzed for the first time in U251 glioblastoma cells. The results provide valuable data on the effects of EGF and bFGF in glioma-derived cancer stem cells.

## Materials and methods

### Cell culture

The human glioblastoma U251 cell line and the human embryonic kidney 293 cell line (HEK 293) (ATCC, Manassas, VA, USA) were used in all experiments and were maintained in Dulbecco’s modified Eagle’s medium (DMEM; Hyclone) supplemented with 10% fetal bovine serum (Hyclone), 1% penicillin-streptomycin solution and 1% GlutaMAX (Sigma, St. Louis, MO, USA). The cells were cultured under standard conditions (humidified atmosphere of 5% CO_2_ in air at 37°C).

### ELISA

Conditioned medium from the U251 cells was collected for the ELISA following incubation for 1, 3 or 5 days. Concentrations of EGF and bFGF in the cell culture supernatant were determined using a Quantikine Human EGF Immunoassay kit (Shanghai Weiao Biotech Ltd., Shanghai, China) and a bFGF Immunoassay kit (Shanghai Weiao Biotech Ltd.), respectively, according to the manufacturer’s instructions.

### Preparation of siRNA and construction and identification of enhanced green fluorescent protein (EGFP)-labeled lentiviral expression vector

Four siRNA duplexes targeting human EGFR (GenBank accession no. NM_201283.1) and bFGFR (GenBank accession no. NM_023107.2) were used. All siRNA duplexes were designed and synthesized by Shanghai R&S Biotechnology Co. Ltd. (Shanghai, China), using 2′-bis(acetoxyethoxy)-methyl ether (ACE) protection chemistry.

The siRNA duplexes were prepared by annealing the complementary oligos and were ligated into the expression vector pcDNA6.2-EGFP (Invitrogen, Carlsbad, CA, USA) to form pcDNA6.2-EGFP-EGFR and pcDNA6.2-EGFP-bFGFR, respectively. The products were used to transform *Escherichia coli* DH5α cells. Following overnight culture of the cells with 50 mg/l spectinomycin, pcDNA6.2-EGFP-EGFR and pcDNA6.2-EGFP-bFGFR were extracted and the sequences of the EGFR and bFGFR siRNA were confirmed by sequencing. The siRNA sequences were screened based on their interference efficiency in HEK 293 cells, which was evaluated by fluorescence microscopy and reverse transcription polymerase chain reaction (RT-PCR). The most efficient duplex was selected for subsequent studies. The target sequences of the siRNAs were 5′-AAGTGTGTAACGGAATAGGTA-3′ for EGFR and 5′-GCAACGTGGAGTTCATGTGTA-3′ for bFGFR.

To construct the lentiviral expression vector, pcDNA6.2-EGFP-EGFR and pcDNA6.2-EGFP-bFGFR were amplified by PCR using a primer pair with *Asc*1 and *Pme*1 sequences (5′-TACTGGCGCGCCGCCACCATGGTGAG CAAGGGCGAGGA-3′ and 5′-ACTAGTTTAAACTGCGGC CAGATCTGGGC-3′). Subsequent to restriction endonuclease digestion with *Asc*1 and *Pme*1, the product was ligated into the lentiviral expression vector, pLenti6.3-MCS/V5 DEST (Invitrogen), which was used to transform *E. coli* DH5α cells. Positive recombinant colonies (named pLenti6.3-EGFP-EGFR-miR and pLenti6.3-EGFP-bFGFR-miR) were screened and identified by PCR, *Asc*1 and *Pme*1 digestion and DNA sequencing. To produce lentiviral particles, the positive recombinant plasmid DNA was extracted from the *E. coli* DH5α cells using a plasmid extraction kit (Axygen Biotechnology Ltd., Hangzhou, China) and mixed with 6 μg pLenti6.3-MCS/V5 DEST and 6 μg plasmid packaging mix for co-transfection of the HEK 293 cells using POLOdeliverer 3000 Transfection Reagent (Shanghai R&S), according to the manufacturer’s instructions. Following incubation for 48–72 h, the lentiviral particle supernatant was collected for the next experiment. Packaging efficiency was assessed using fluorescence microscopy.

### Transfection of siRNAs

Exponentially growing U251 cells were plated at a density of 1×10^5^ cells/dish and incubated in 5% CO_2_ at 37°C. At 80% confluence, the cells were divided into five groups [RNA interference (RNAi)-EGFR, RNAi-bFGFR, RNAi-EGFR + bFGFR, RNAi-negative control (NC) and control] and transfected with Lenti-EGFP-EGFR-microRNA (miR), Lenti-EGFP-bFGFR-miR, Lenti-EGFP-EGFR-miR plus Lenti-EGFP-bFGFR-miR, Lenti-GFP and blank DMEM, respectively (multiplicity of infection, MOI = 10). To intensify infection, polybrene (4–8 μg/ml final concentration) was also used. Subsequent to 24 h of incubation, the medium was replaced with fresh DMEM and the U251 cells were incubated for another 48 h. Infection efficiency was assessed by fluorescence microscopy based on the fluorescence of the reporter gene, GFP.

### RNA isolation and cDNA synthesis

Total RNA was extracted from the cultured cells with TRIzol reagent (Invitrogen). cDNA was synthesized using Moloney’s murine leukemia virus (M-MLV; Fermentas, Waltham, MA, USA) in a 20-μl reaction volume containing 1 μg total RNA, 1 μl random primer (0.5 μg/μl), 1 μl oligo(dT) primer (0.5 μg/μl) and RNase-free water. The reaction was incubated at 65°C for 10 min and quickly chilled on ice. Next, 4 μl 5X reaction buffer, 0.5 μl RiboLock ribonuclease inhibitor (20 U/μl), 2 μl 10 mM dNTP mix (Majorbio Bio-Pharm Technology Co., Ltd., Shanghai, China) and 1 μl of ReverTra Ace (Fermentas) was added. The mixture was incubated at 37°C for 60 min. The process was stopped by heating the solution to 70°C for 10 min and then quickly chilling it on ice.

### Quantitative (q)PCR

EGFR, bFGFR, CD133, GFAP, TUBB4 and MBP mRNA was detected by qPCR using the primers listed in Table I. PCR amplification was performed in a 20-μl volume that contained 1 μl reverse transcriptase (Fermentas), 10 μl 2X SYBR Green mix (Invitrogen), 8 μl 10X buffer and 1 μl primers. PCR was performed using an Eppendorf Realplex (Eppendorff, Hamberg, Germany) with initial denaturation at 95°C for 2 min, followed by 40 cycles of 95°C for 15 sec, 59°C for 15 sec and 72°C for 20 sec. The dissociation of the reaction products was conducted from 60 to 95°C for 30 sec with 39 cycles. Fluorescence data were converted into cycle threshold (CT) measurements. The CT of each sample was averaged and analyzed by a comparative CT method. The expression level of a specific gene was determined quantitatively by calculating the ratio relative to an internal standard [human β-actin (hACTB)] as 2^−ΔΔT^.

### Statistical analysis

Statistical analyses were performed using SPSS software (version 11.5.0 for Windows; SPSS, Inc., Chicago, IL, USA). Statistical significance was evaluated with Student’s t-test, using data from at least three independent experiments. Values of P<0.05 were considered to indicate a statistically significant difference.

## Results

### Confirmation of recombinant lentiviral expression vectors

**Figure 1:**
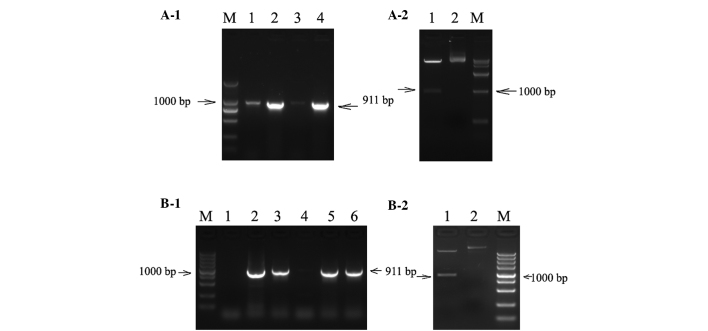
Identification of EGFP-labeled lentiviral expression vector. (A-1) Gel electrophoresis of the PCR product of pLenti6.3-EGFP-EGFR-miR. M, marker DL2000; lane 1, negative control; lanes 2–4, PCR product of recombinant vector (911 bp). (A-2) Restriction map of the PCR positive vector of pLenti6.3-EGFP-EGFR-miR. M, marker DL15000; lane 1, digestion product of double restriction endonuclease of *Asc*I and *Pme*I; lane 2, the recombinant plasmid pLenti6.3-EGFR-EGFR-miR. (B-1) Gel electrophoresis of the PCR product of pLenti6.3-EGFP-FGFR-miR. M, marker DL5000; lane 1, negative control; lanes 2–6, PCR product of recombinant vector (911 bp). (B-2) Restriction map of the PCR positive vector of pLenti6.3-EGFP-FGFR-miR. M, marker DL5000; lane 1, digestion product of double restriction endonuclease of AscI and PmeI; lane 2, the recombinant plasmid pLenti6.3-EGFP-FGFR-miR. EGFP, enhanced green fluorescent protein; EGFR, epithelial growth factor receptor; miR, microRNA; PCR, polymerase chain reaction; FGFR, fibroblast growth factor receptor.

Following transformation with the lentiviral expression plasmids, pLenti6.3-EGFP-EGFR-miR and pLenti6.3-EGFP-bFGFR-miR, the *E. coli* DH5α cells were grown in oscillating culture and spread onto ampicillin-lysogeny broth plates. Single clones were selected for PCR amplification. Agarose gel electrophoresis of the PCR products showed the expected 911-bp insert, and digestion with *Asc*1 and *Pme*1 yielded the appropriate fragments (Fig. 1). Thus, the recombinant lentiviral expression vectors were constructed successfully.

**Figure 2:**
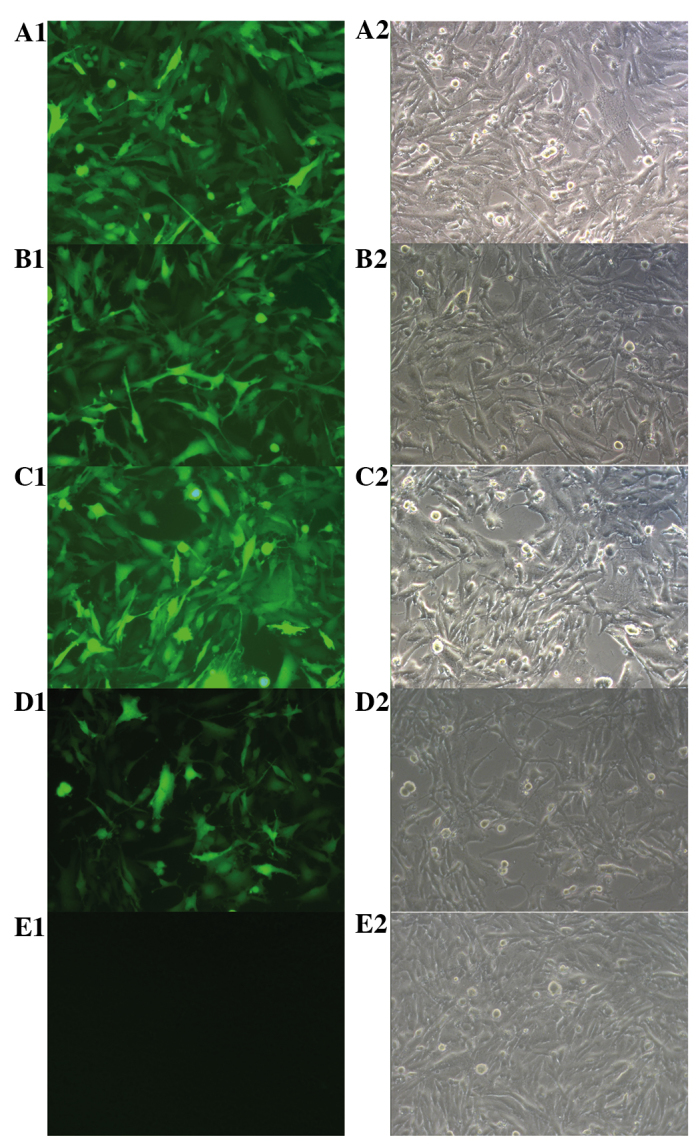
Fluorescence microscope map of U251 cells. U251 cells display green fluorescence after transfection with (A1) Lenti-EGFP-EGFR-miR, (B1) Lenti-EGFP-bFGFR-miR, (C1) Lenti-EGFP-EGFR-miR+Lenti-EGFP-bFGFR-miR, or (D1) Lenti-EGFP at 60 h. (E1) Blank control (A2, B2, C2, D2 and E2): phase contrast microscope (Original magnification, ×400). EGFP, enhanced green fluorescent protein; EGFR, epithelial growth factor receptor; miR, microRNA; bFGFR, basic fibroblast growth factor receptor.

**Figure 3:**
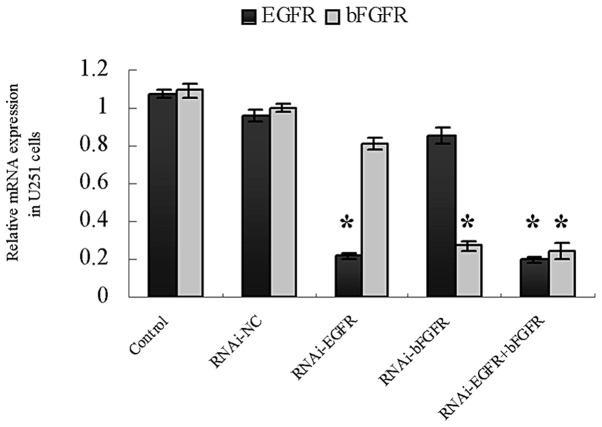
Expression of EGFR and bFGFR in U251 cells evaluated by quantitative polymerase chain reaction (qPCR). A decrease in EGFR mRNA expression was observed in U251 cells after transfection with Lenti-EGFP-EGFR-miR and Lenti-GFP-EGFR-miR + Lenti-EGFP-FGFR-miR [comparison with cell control and RNAi-negative control (NC); P<0.005]. The downregulated mRNA expression of bFGFR was also detected in U251 cells transfected with Lenti-EGFP-FGFR-miR and Lenti-EGFP-EGFR-miR + Lenti-EGFP-FGFR-miR (^*^P<0.005; comparison with cell control and RNAi-NC). EGFP, enhanced green fluorescent protein; EGFR, epithelial growth factor receptor; miR, microRNA; bFGFR, basic fibroblast growth factor receptor.

Selected EGFR and FGFR siRNA sequences markedly decreased target expression in the U251 cells. Target mRNA expression in the U251 cells was assayed 72 h after transfection with the lentiviral particles carrying the siRNA sequences (Lenti-EGFP-EGFR-miR and/or Lenti-EGFP-bFGFR-miR). Transfection was confirmed by the expression of EGFP in the U251 cells, as assessed by fluorescence microscopy (Fig. 2). No green fluorescence was observed in the non-transfected U251 control cells. The expression levels of EGFR and bFGFR mRNA was analyzed by qPCR. The mRNA expression of EGFR and bFGFR was significantly reduced compared with the expression in the control cells (P<0.01). The siRNA sequences targeting EGFR and bFGFR effectively knocked down the mRNA levels of these receptors by at least 80% (Fig. 3).

### U251 cells express EGF/EGFR and bFGF/bFGFR

**Figure 4:**
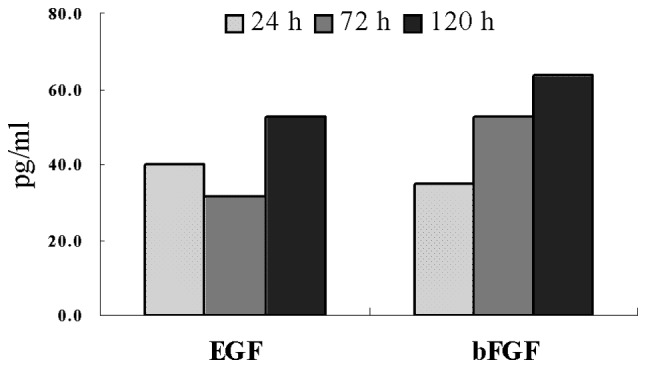
Concentration of EGF and bFGF in U251 cell culture supernatant, as detected by ELISA. EGF, epithelial growth factor; bFGF, basic fibroblast growth factor.

The expression of EGF/bFGF and EGFR/bFGFR in the U251 cells was examined. qPCR showed that the EGFR and bFGFR mRNA was expressed in the U251 cells (Fig. 3). To determine the expression of EGF and bFGF, ELISAs of the U251 cell culture supernatants were performed. The levels of EGF and bFGF in the supernatants increased with the culture time. At 5 days, the concentrations of EGF and bFGF were 52.2 and 63.4 pg/ml, respectively (Fig. 4).

### CD133, GFAP, MBP and TUBB3 mRNA expression in U251 cells with EGFR and bFGFR gene knockdown

**Figure 5:**
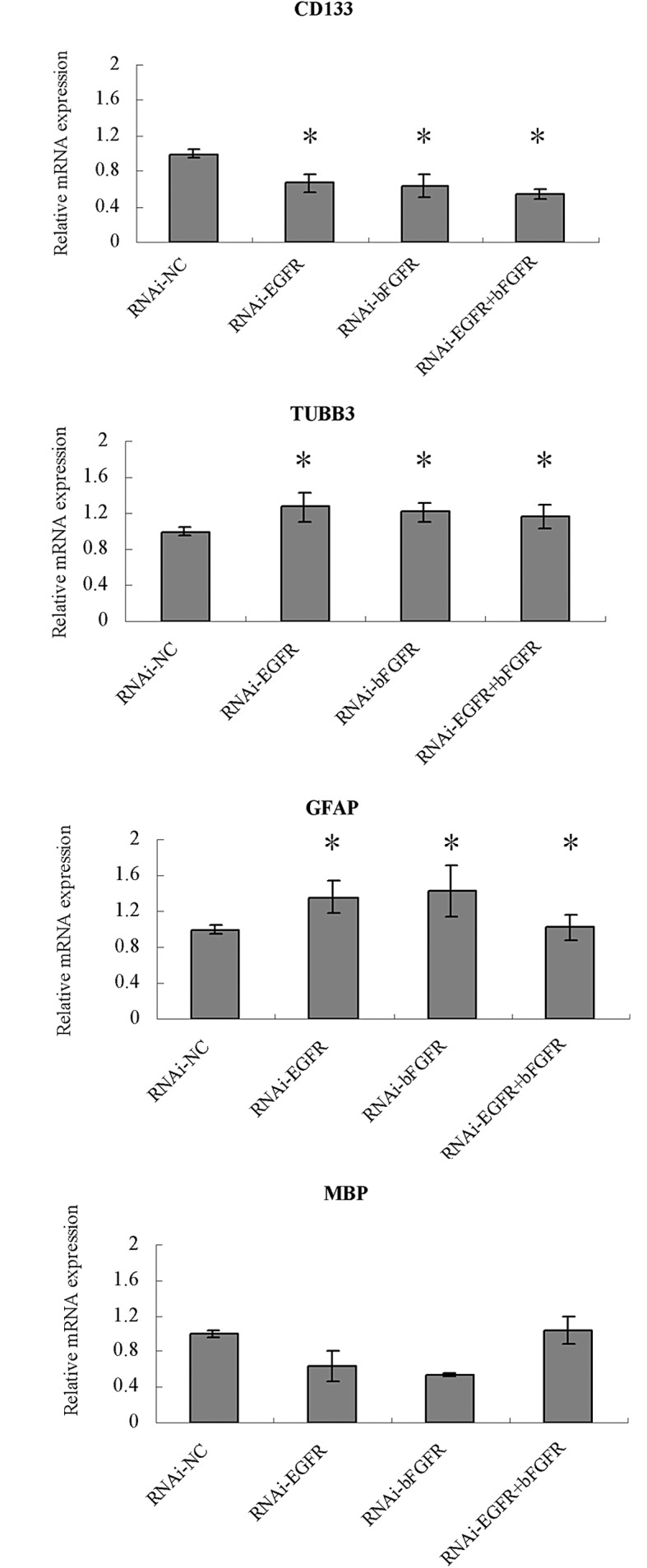
Relative mRNA expression of CD133, TUBB3, GFAP and MBP in U251 cells measured by qPCR. U251 cells were transfected with Lenti-EGFP-EGFR-miR or Lenti-EGFP-FGFR-miR, or both Lenti-EGFP-EGFR-miR and Lenti-EGFP-FGFR-miR. U251 cells transfected with Lenti-EGFP were set up as control groups. ^*^P<0.05. CD133, cluster of differentiation 133; TUBB3, tubulin-β3; GFAP, glial fibrillary acidic protein; MBP, myeline basic protein; EGFP, enhanced green fluorescent protein; EGFR, epithelial growth factor receptor; miR, microRNA; bFGFR, basic fibroblast growth factor receptor; RNAi, RNA interference; NC, negative control; qPCR, quantitative polymerase chain reaction.

To evaluate mRNA expression levels of the genes of interest, qPCR was performed once the U251 cells had been transfected with Lenti-EGFP-EGFR-miR, Lenti-EGFP-FGFR-miR or both. The results are shown in Fig. 5. Non-transfected U251 control cells expressed CD133, GFAP, MBP2 and TUBB3 mRNA. After silencing EGFR and/or bFGFR expression, the mRNA expression levels of CD133, GFAP, MBP and TUBB3 were altered. CD133 mRNA expression was significantly reduced in the RNAi-EGFR, RNAi-bFGFR and RNAi-EGFR + RNAi-FGFR groups compared with that in the control (RNAi-NC) group (P<0.05), with particularly marked downregulation in the RNAi-EGFR + RNAi-FGFR group. The mRNA expression levels of TUBB3 and GFAP in the U251 cells were significantly increased in the RNAi-EGFR, RNAi-bFGFR and RNAi-EGFR + RNAi-FGFR groups compared with those in the RNAi-NC group (P<0.05). However, the mRNA expression level of CD133, GFAP and TUBB3 did not differ significantly among the RNAi-EGFR, RNAi-bFGFR and RNAi-EGFR + RNAi-FGFR groups. The expression of the MBP mRNA was decreased in the RNAi-EGFR and RNAi-bFGFR groups, but was increased in the RNAi-EGFR + RNAi-FGFR group; however, the MBP mRNA level in the RNAi-EGFR + RNAi-FGFR group was not significantly different from that in the RNAi-NC group.

## Discussion

Glioblastoma, the most common and malignant astrocytoma, is believed to have a glial cell origin. GFAP, a specific marker for immature or neoplastic astrocytes, is usually expressed in glioblastoma cells (10). The present study assessed the expression of a stem cell marker (CD133), an astrocyte marker (GFAP), a neuronal marker (TUBB4) and an oligodendrocyte marker (MBP) using qPCR. The U251 cells expressed CD133 mRNA, which is consistent with our previous observations using immunohistochemistry and PCR in different WHO-grade astrocytomas (11). Co-expression of the neuronal (TUBB4) and oligodendrocytic (MBP) markers was also observed in the U251 cells. These results indicate that multilineage phenotypes exist in U251 cells. Stem cell markers and multilineage phenotypes have also been described in glioblastomas (12). The expression of neuronal, astrocytic and oligodendroglial lineage markers indicates that U251 glioblastoma cells are multipotent or plastic cells. This multilineage phenotype may be a consequence of the differentiation of glioblastoma cells under environmental regulation (13).

Determining the differentiation mechanism(s) of glioblastoma cells may help in developing opportunities for interfering with the glioblastoma phenotype, which may be useful in treating glioblastomas (14). Various autocrine factors found in adult tissue and malignant tumors provide growth signals. Previous studies have demonstrated the overexpression of EGFR and FGFR, and their co-expression with their ligands occurs frequently in gliomas (1–3). These studies indicate that EGFR and FGFR are candidate receptors for autocrine growth factors in gliomas.

In the present study, mRNA expression of EGFR and bFGFR was detected in U251 glioblastoma cells, and EGF and FGF were detected in U251 cell supernatants. These results indicate that active EGF-EGFR and FGF-FGFR autocrine pathways exist in U251 cells. It has been reported that EGF and bFGF are critical components of human embryonic stem cell and cancer stem cell culture media, and that they are crucial for maintaining glioma stem cells in an undifferentiated state (15–17). The EGF-EGFR and FGF-FGFR autocrine pathways may be involved in the dedifferentiation mechanism(s) of glioblastoma cells.

siRNA mediates sequence-specific mRNA degradation and has become a useful tool for silencing specific target genes. In the present study, siRNA duplexes targeting human EGFR and bFGFR were designed, synthesized and used to knock down EGFR and/or FGFR gene expression, in order to aid in the elucidation of the roles of EGF-EGFR and FGF-FGFR autocrine pathways in the differentiation of U251 glioblastoma cells. EGFP-labeled recombinant lentiviral expression vectors for the siRNAs were constructed and confirmed by PCR, restriction enzyme digestion and DNA sequencing. The siRNAs targeting EGFR and FGFR effectively knocked down the receptor mRNA levels in the U251 cells.

EGF and bFGF are important autocrine factors in glioblastomas and enable cancer stem-like cells to persist in established glioblastoma cell lines without the addition of exogenous growth factors (18,19). Activated EGFR and bFGFR signaling increases cell proliferation, survival and migration, and blocks glial and neuronal differentiation in glioblastomas. In the present study, knockdown of the EGFR and/or bFGFR genes significantly downregulated CD133 mRNA expression and facilitated U251 cell glial and neuronal differentiation. Knockdown of both EGFR and FGFR appeared to act synergistically to downregulate CD133 expression. This demonstrates the importance of EGF and bFGF in maintaining the undifferentiated state of cancer stem cells and stem cells. However, this is not the case for promoting glial and neuronal differentiation of U251 cells. In fact, the facilitation of U251 cell glial and neuronal differentiation was almost the same whether the EGFR pathway, the FGFR pathway or both pathways were blocked. Notably, knocking down EGFR and/or FGFR did not facilitate oligodendrocyte differentiation of the U251 cells, as others have reported in stem cells (20). By contrast, MBP mRNA expression was downregulated subsequent to blocking either the EGFR or FGFR pathway. These findings indicate that EGFR and/or FGFR contribute to the regulation of stem cell marker gene expression and differentiation.

Stem cell markers and multilineage phenotypes have been described in glioblastomas. The present study showed that U251 glioblastoma cells express stem cell markers and neuronal, astrocytic and oligodendroglial lineage markers. EGFR and bFGFR mRNA and EGF and bFGF proteins were detected in U251 cells and culture supernatant, respectively. This indicated that autocrine and/or paracrine EGF and/or bFGF mechanisms exist in U251 cells. The knockdown of the EGFR and/or FGFR genes downregulated CD133 mRNA expression and facilitated glial and neuronal differentiation in the U251 cells.

## Figures and Tables

**Table I:** Sequences of PCR primers used in the study.

Gene name	Forward primer	Reverse primer	Size, bp
EGFR	TGACTGAGGACAGCATAGACGA	GGGCTGGACAGTGTTGAGATAC	203
FGFR	GGCTGCCAAGACAGTGAAGTT	GGTTGATGCTGCCGTACTCAT	215
GFAP	GCACGCAGTATGAGGCAATG	ACTCCAGGTCGCAGGTCAAG	177
TUBB3	CCAAGGGTCACTACACGGAG	ATGATGCGGTCGGGATACTC	187
CD133	CCGCAGGAGTGAATCTTTTATC	CCATTCCCTGTGCGTTGA	199
MBP2	CCGAGAAGGCCAGTACGA	GTGAAAGTTCACCCAGGTTTCT	74
hACTB	TCCTTCCTGGGCATGGAGT	CAGGAGGAGCAATGATCTTGAT	208

EGFR, epithelial growth factor receptor; FGFR, fibroblast growth factor receptor; GFAP, glial fibrillary acidic protein; TUBB3, tubulin-β3; CD133, cluster of differentiation 133; MBP2, myelin basic protein 2; hACTB, human β-actin.

## Abbreviations

EGF: epithelial growth factor
bFGF: basic fibroblast growth factor
EGFR: epithelial growth factor receptor
bFGFR: basic fibroblast growth factor
GFAP: glial fibrillary acidic protein
HEK293: human embryonic kidney cell line 293
DMEM: Dulbecco’s modified Eagle’s medium
FBS: fetal bovine serum
